# Blue Ear Cyst: A Rare Eccrine Hidrocystoma of the Ear Canal and Successful Endoscopic Excision

**DOI:** 10.1155/2016/5902547

**Published:** 2016-11-06

**Authors:** Taha A. Mur, Ronald Miick, Natasha Pollak

**Affiliations:** ^1^Lewis Katz School of Medicine, Temple University, Philadelphia, PA, USA; ^2^Department of Pathology and Laboratory Medicine, Einstein Medical Center, Philadelphia, PA, USA; ^3^Department of Otolaryngology-Head & Neck Surgery, Lewis Katz School of Medicine, Temple University, Philadelphia, PA, USA

## Abstract

*Aims*. Hidrocystomas are benign cystic growths of the apocrine and eccrine sweat glands. These cystic lesions have been well documented on the face, head, and neck, but rarely in the external auditory canal.* Presentation of Case*. A 67-year-old woman presented with a bluish cystic mass partially occluding the external auditory canal and interfering with hearing aid use. Lesion was excised completely via a transcanal endoscopic approach with excellent cosmetic results, no canal stenosis, and no recurrence at 1-year follow-up.* Discussion*. We present a rare eccrine hidrocystoma of the external auditory canal and successful excision of this benign lesion. We describe the surgical management using a transcanal endoscopic approach and follow-up results. An eccrine gland cyst that presents as a mass occluding the external auditory canal is quite rare. There are only a few such cases reported in the literature. These masses can be mistaken for basal cell carcinomas or cholesterol granulomas but can be easily differentiated using histopathology.* Conclusion*. Eccrine hidrocystoma is a cystic lesion of sweat glands, rarely found in the external auditory canal. A characteristic bluish hue aids in diagnosis and surgical excision using ear endoscopy provides excellent control.

## 1. Introduction

The skin of the external auditory canal, just like skin elsewhere in the body, contains several types of adnexal secretory glands, including eccrine (common sweat glands), apocrine (modified sweat glands), and holocrine (sebaceous) glands. The external canal skin also contains ceruminous glands, essentially apocrine glands, which, in combination with the sebaceous glands, create cerumen [1]. The balance of secretory functions creates a slightly acidic (pH 6) microenvironment in the ear canal that supports a balanced and healthy skin flora.

Several types of solid tumors have been reported to originate from these glands, both benign, such as ceruminous adenomas, and malignant, such as ceruminous adenocarcinomas [2]. Cystic masses originating from these secretory glands are rarely seen in the external auditory canal (EAC). Cystic lesions associated with the apocrine glands have been documented in the literature to occur within the eyelid, axillae, and groin [2]. These cysts, also known as apocrine hidrocystomas, occasionally present as bluish masses [3, 4]. Similarly, cystic tumors associated with the eccrine glands or eccrine hidrocystomas are extremely rare in the ear canal, with only a few cases reported in the literature [1, 2, 4].

Here, we present a rare case of an eccrine hidrocystoma, confined to the external auditory canal in an adult patient. The lesion was surgically excised using endoscopic ear surgery techniques. There was no recurrence, EAC stenosis, or other complications.

## 2. Presentation of Case

**Figure 1:**
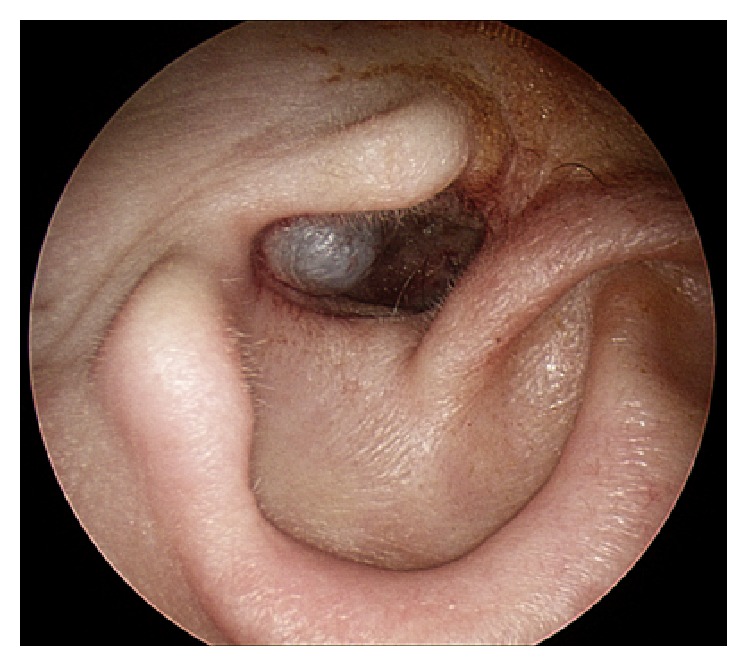
A bluish lesion is seen in the external auditory canal inferiorly, partially blocking the canal lumen.

A 67-year-old woman presented to our otolaryngology clinic complaining of a lesion in her left ear canal that had been growing slowly for approximately 10 years. She had prior bilateral mastoidectomies (intact canal wall) for chronic otitis media as a child. Over the past 20+ years, her ear symptoms have been quiescent and there is no ongoing inflammatory process in her ears. She has some mixed hearing loss and wears bilateral hearing aids. Recently, the lesion in her left ear canal has interfered with hearing aid placement. On otoscopic examination, a soft, nontender, ovoid, smooth, bluish mass about 1 cm in diameter was noted partially blocking the left EAC meatus (Figure 1).

Her tympanic membrane, which could still be partially visualized past the lesion, appeared grossly intact with normal landmarks. Audiometry was consistent with a severe to profound mixed hearing loss bilaterally. Tympanometry was normal (type A) on the right, but on the left ear, a seal could not be achieved.

**Figure 2:**
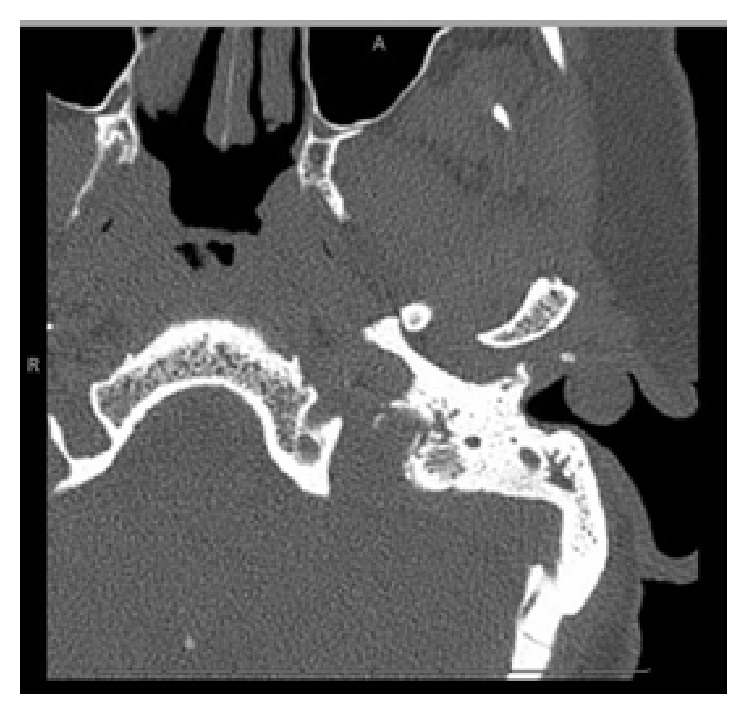
CT of the temporal bones shows a well-defined cystic lesion originating from the anterolateral portion of the left external auditory canal. The lesion has no deep extension, does not show any bone erosion, and does not involve the middle ear cleft.

To evaluate the deep extent of the lesion, the patient underwent a CT scan of the temporal bones without contrast, which revealed a well-circumscribed 1.0 × 0.8 cm sessile cystic lesion arising from the floor of the lateral portion of the left external auditory canal. A thin rim of calcification could be seen along the inferior margin of the lesion. There was no evidence of invasion of surrounding soft tissues or bone. The tympanic membrane, middle ear cleft, and ossicular chain were intact and normal. Prior mastoidectomies were evident on CT images (Figure 2).

**Figure 3:**
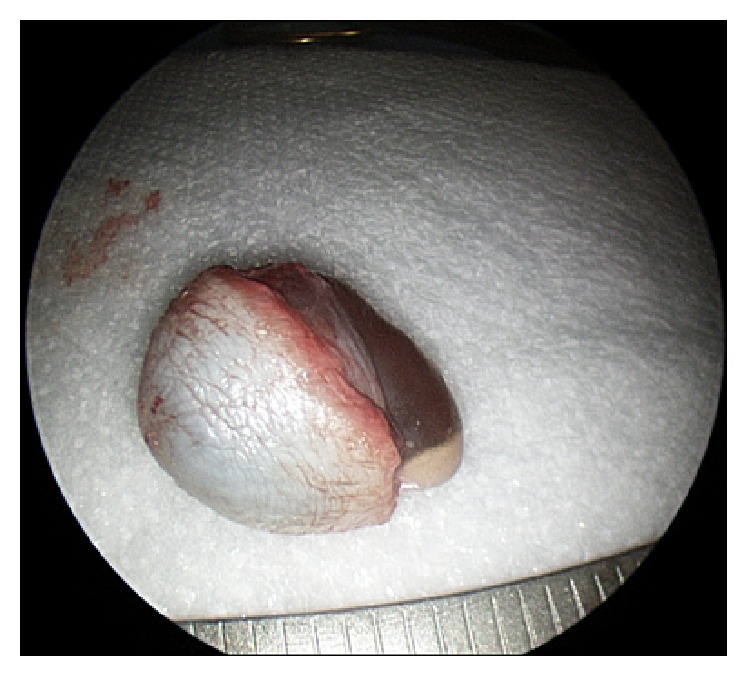
A bluish smooth cystic lesion is excised with some overlying skin. The lesion contains brown liquid.

Because of the benign features of the lesion on history, examination, and imaging, decision was made to proceed with local excision of the lesion with minimal margins. The patient was taken to the operating room for excision of this lesion under general anesthesia, using the otologic endoscope and binocular otomicroscopy. The mass was completely enucleated via a transcanal approach. The lesion was broadly attached to the anteroinferior surface of the external auditory canal but did not involve the bony canal or the tragal cartilage. Minimal bleeding was encountered which was controlled with simple pressure. No skin closure was needed for the resultant defect of less than 10 mm diameter. Bacitracin ointment was applied to the wound. After excision, the bluish smooth cystic lesion was opened and dark brown liquid was seen emanating from the lumen (Figure 3).

**Figure 4:**
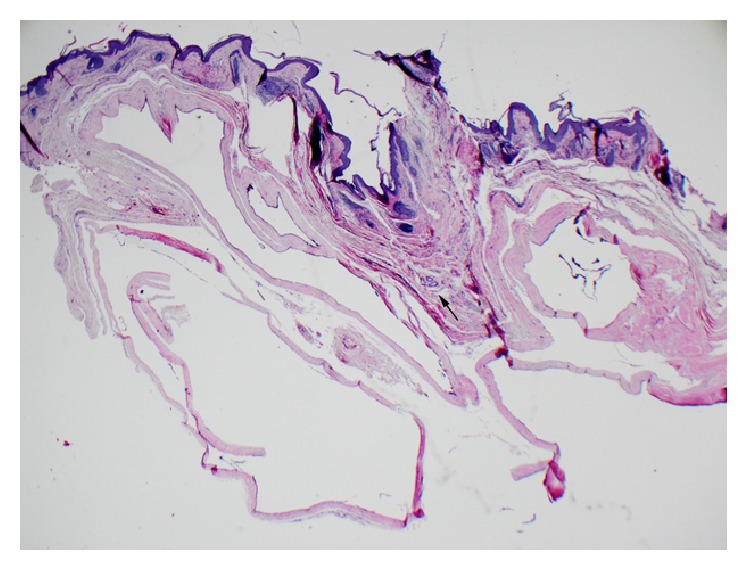
Histopathologic examination shows external auditory canal skin with multiple dermal cysts. A focus of normal sweat glands is present between the cysts in the middle of the photomicrograph (arrow).* Hematoxylin and eosin stain, 20x.*

**Figure 5:**
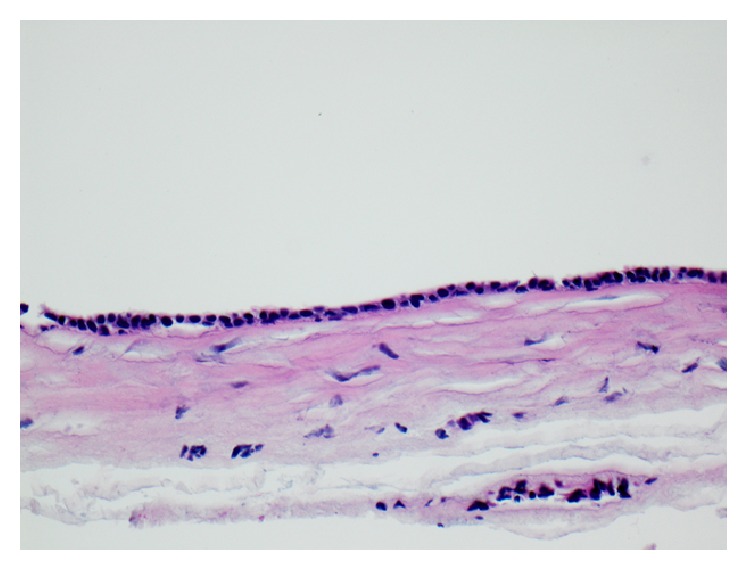
Close-up of a cyst wall demonstrates a cuboidal double-layered epithelium, consistent with an eccrine hidrocystoma.* Hematoxylin and eosin stain, 400x. *

Histopathological examination showed skin with multiple small dermal cysts ranging from 2 to 5 millimeters, with scattered normal sweat glands present between the cysts (Figure 4). On higher magnification, the cysts are lined by a flattened cuboidal double-layered epithelium. Apocrine features, such as columnar cell epithelium with dense eosinophilic cytoplasm and decapitation secretion, are not identified (Figure 5). The histologic features are consistent with an eccrine hidrocystoma.

The patient did well postoperatively with no complications. A postoperative audiogram revealed stable hearing and normal tympanometry (type A) bilaterally. The wound site epithelialized well and the surgical site healed without scarring or stenosis. The lesion did not recur during the 12-month follow-up period. The patient was now able to use her hearing aids without difficulty.

## 3. Discussion

Hidrocystomas of the external ear canal are rare, benign cystic lesions and are generally categorized as apocrine or eccrine [3, 5]. They usually present as papular or nodular firm cystic lesions with a smooth surface and bluish appearance. The clinical presentation can be variable and is dependent on the lesion's anatomic location and impact on surrounding structures. These masses can obstruct the lumen of the ear canal, interfere with hearing aid use, and may be mistaken for basal cell carcinomas or cholesterol granulomas but can be easily differentiated from the others by examining their histopathologic features. As in a cholesterol granuloma, the bluish hue is an optical illusion. The cystic space of an eccrine hidrocystoma contains either clear or brown fluid. The presence of multiple hidrocystomas may be associated with focal dermal hypoplasia, a genetic disorder, formerly referred to as Jessner-Cole syndrome or Goltz-Gorlin syndrome. Our patient had a solitary hidrocystoma.

Eccrine hidrocystomas are characterized by the presence of cysts lined with attenuated double-layered epithelium which lack features of apocrine cell differentiation, such as decapitation secretion and tall columnar cells with eosinophilic cytoplasm. Scattered benign sweat glands are often found admixed with the cystic glands. Some authors suggest that eccrine hidrocystomas may actually be of apocrine type, with the typical apocrine features attenuated due to the intraluminal pressure of the cyst fluid [6].

Surgical excision is the treatment of choice. In patients with multiple hidrocystomas, or lesions that are not easily accessible to surgery, alternative treatments exist, such as topical atropine, scopolamine cream, botulinum toxin injection, or CO_2_ laser ablation [7]. The choice of surgical approach is largely dictated by the tumor location and relationship to surrounding structures. For lesions limited to the external auditory canal, without involvement of the tympanic membrane or mastoid, endoscopic transcanal approaches are becoming increasingly popular. The advantage of an endoscopic approach is superior visualization of the tumor due to the wide-angle view provided by the Hopkins rod endoscope. In our patient, the mass was completely surgically excised with endoscopic assistance, and the patency of the EAC was maintained without any surgical complications or recurrences.

## 4. Conclusion

While hidrocystomas are generally uncommon cystic lesions of the sweat glands, it is quite rare to find an eccrine hidrocystoma originating from the skin of the external auditory canal. The slow growing nature and smooth, solitary, cystic appearance, in addition to the bluish tint, can provide valuable diagnostic clues [3]. Complete excision with no margins is curative and can be done via a transcanal endoscopic approach.

## References

[1] Stoeckelhuber M., Matthias C., Andratschke M. (2006). Human ceruminous gland: ultrastructure and histochemical analysis of antimicrobial and cytoskeletal components. *Anatomical Record—Part A Discoveries in Molecular, Cellular, and Evolutionary Biology*.

[2] Obaidat N. A., Ghazarian D. M. (2006). Bilateral multiple axillary apocrine hidrocystomas associated with benign apocrine hyperplasia. *Journal of Clinical Pathology*.

[3] Sarabi K., Khachemoune A. (2006). Hidrocystomas—a brief review. *MedGenMed Medscape General Medicine*.

[4] Haro-García M., Corzón-Pereira T., Morales-Puebla J. M., Figueroa-García T. (2015). Eccrine hidrocystoma of the external auditory canal. *Acta Otorrinolaringologica Espanola*.

[5] Ioannidis D. G., Drivas E. I., Papadakis C. E., Feritsian A., Bizakis J. G., Skoulakis C. E. (2009). Hidrocystoma of the external auditory canal: a case report. *Cases Journal*.

[6] Weedon D. (2010). *Weedon's Skin Pathology*.

[7] Lee M. R., Ryman W. (2004). Multiple eccrine hidrocystomas. *Australasian Journal of Dermatology*.

